# Accelerating stable recombinant cell line development by targeted integration

**DOI:** 10.1186/1753-6561-7-S6-P111

**Published:** 2013-12-04

**Authors:** Bernd Rehberger, Claas Wodarczyk, Britta Reichenbächer, Janet Köhler, Renée Weber, Dethardt Müller

**Affiliations:** 1 Rentschler Biotechnologie GmbH, 88471 Laupheim, Germany

## Introduction

Targeted integration (TI) allows fast and reproducible genetic modification of well characterized previously tagged host cells thus generating producer cells with predictable qualities. Concurrently, timelines are cut by 50% compared to random integration (RI) based cell line development. In contrast to commonly low productivities of cell lines generated by TI, we developed a system for CHO cells leading to productivities of more than 1 g/L within weeks using the TurboCell™ platform.

The system is based on CHO K1 cells that have been tagged with a GFP expression cassette flanked by recombinase recognition sites. Following GFP based FACS enrichment and cloning of the tagged cells, over 4000 clones were screened for growth, productivity, GFP expression stability and integration status of the GFP expression cassette. The best clones were selected to be used as "Master TurboCell" (MTC) host cell lines for recombinant cell line development.

## Generation of producer TurboCell™ lines

A selected MTC host cell line is co-transfected using a TurboCell™ expression plasmid containing the gene of interest (GOI) expression cassette flanked by matching recombinase recognition sites together with a plasmid encoding the recombinase enzyme required for RMCE. Upon transfection both plasmids enter the MTC's nucleus initiating transient expression of the recombinase which further mediates the stable exchange of the GFP expression cassette against the GOI expression cassette. Thus, the GOI is stably introduced into the tagged genomic spot shortly after the transfection. Cells are cultivated for a few days to recover from the transfection procedure and to allow GFP to fade out of RMCE positive cells. The transfected pools are thereupon sorted by FACS in order to remove the majority of GFP positive cells. The remaining producer TurboCell™(PTC) pools in general comprise of 90-99% GFP negative, GOI expressing cells that are genetically identical due to the conserved locus of GOI integration. This allows the production of recombinant protein from PTC enriched pools at a very early stage of 3 weeks upon transfection. Due to their genetic homogeneity the physiological diversity of the clones within the pool is limited thus leading to only small variations in the recombinant protein produced. Therefore, material drug candidate screening prepared on the parental PTC pool level should only differ slightly from material produced from clones thereof. Following FACS sorting, the PTC pools can be cloned, if required. Due to the high degree of similarity of all clones, the screening effort to find the best clone can be limited to about 10 clones. Recombinant protein material from clones can be produced 9 weeks upon transfection.

## Molecular biological analysis of producer TurboCell™ lines

In order to prove successful RMCE reproducibly taking place without additional random integration of the remaining plasmid, genomic DNA was prepared from clonal PTC for Southern Blot analysis. The genomic DNA was digested with a restriction enzyme cutting the correctly integrated targeting vector into two pieces, one fragment only comprising internal vector sequences, as well as a second fragment also comprising CHO derived sequences of the specific integration locus. As both fragments carry sequences of the CMV promotor, both can be visualized using one single CMV promoter-specific probe. As only two bands occur in case of successful RMCE, cell lines showing more than two bands indicate clones with randomly integrated targeting vector molecules in addition to RMCE. Statistics of several cell line generation projects show that in about 90% of all analyzed clones a correct RMCE without additional random integration events takes place. This allows for a significant reduction in clone screening efforts to a level of 10 clones per project.

## Process characteristics

**Figure 1a:**
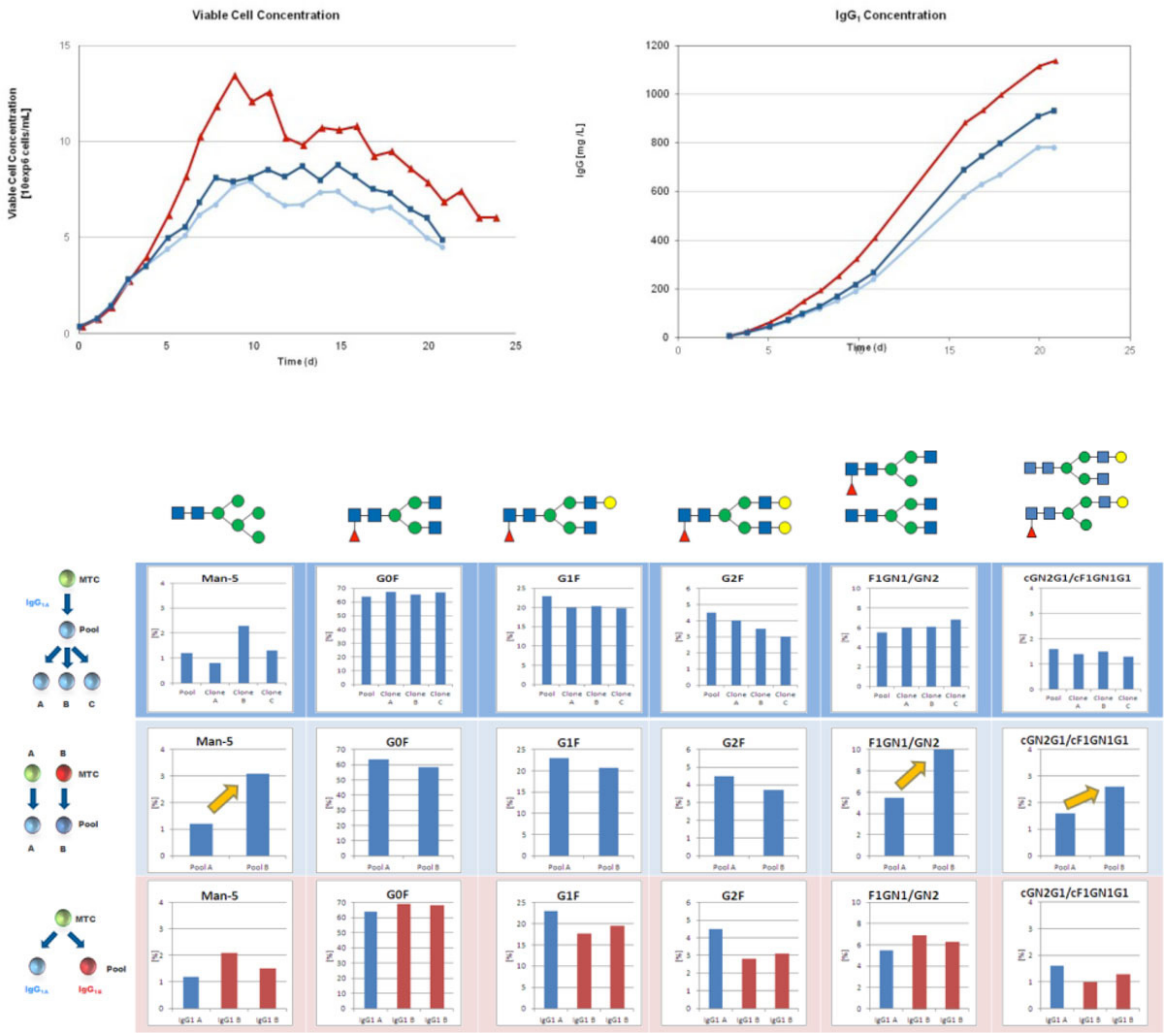
**Comparison of cell growth and recombinant protein production in a bioreactor versus shake flask**. **Figure 1b: **Comparison of glycopatterns. The first row compares three clones derived from one parental pool of one defined MTC with each other and with the relevant parental pool. The second row compares mAb material derived from two PTC pools derived from different, not related MTCs. The third row compares two types of IgG1 antibodies expressed from PTC pools derived from the same MTC.

To show the feasibility of the TurboCell™ system for recombinant protein production in fed batch cultivations, a PTC clone producing IgG1 was cultivated in a stirred tank bioreactor. The data of this bioreactor were compared to two shake flask fed batch runs performed with the same PTC and the same media system (Rentschler's proprietary media + GE Healthcare's ActiCHO feed) in parallel. Figure 1a shows a comparison of viable cell density and product concentration. The better performance in the bioreactor indicates that the PTC can be easily transferred from shake flask to bioreactor settings. The maximum cell density of 13*10^6 ^cells/mL, as well as the integral of viable cells over the cultivation time, and the maximum product titer of more than 1 g/L IgG1 proved the feasibility of the TurboCell™ system for the production of recombinant proteins even in larger amounts at a very early stage of a biopharmaceutical development project.

## Analysis of Protein Quality

Both amount of glycosylation and glycosylation pattern in different Turbo Cell subsets were analyzed

Figure 1b shows that clones derived from the same parental pool differ only slightly regarding their glyco pattern and they are very similar to their parental pool. Even if different antibodies are expressed from pools derived from the same MTC the variation between the pools is within the range of clones compared to each other. Significant variations in the glyco pattern can only be detected, if antibody material derived from pools descendent from different, unrelated MTCs is compared (indicated by yellow arrows).

## Conclusions

Within 3 weeks upon transfection and targeted integration, producer cell pools were FACS sorted to purities of >95%. These cells were suited for high quality recombinant protein material production in fed batch runs exceeding 1 g/L IgG1. Clones generated thereof behaved similar to the pools in terms of productivity and product quality, cell growth and metabolism. From those clones analyzed a mean of about 90% showed successful RMCE without unintended random integration. Cellular properties and productivities of the clones were as expected and variations between different clones were marginal. Thus, the TurboCell™ system reduces clone screening efforts to a minimum allowing the simultaneous production of multiple recombinant proteins in stable CHO cells with optimal use of resources. This makes the TurboCell™ system an interesting tool for candidate screening and early phases material production even in large scale setups.

